# The Effect of Dopamine Secreted by the Brain into the Systemic Circulation on Prolactin Synthesis by the Pituitary gland in Ontogenesis

**Published:** 2016

**Authors:** Yu.O. Nikishina, A.Ya. Sapronova, M.V. Ugrumov

**Affiliations:** Koltsov Institute of Developmental Biology RAS, 26 Vavilov str., Moscow, 119334, Russia; National Research University “Higher School of Economics”, 20 Myasnitskaya str., Moscow, 101000 , Russia

**Keywords:** dopamine, prolactin, brain, pituitary gland, blood-brain barrier, ontogenesis

## Abstract

This research was aimed at studying the brain’s endocrine function in ontogenesis. It has been previously shown in our laboratory that the brain serves as the source of dopamine in the systemic circulation of rats prior to the formation of the blood-brain barrier. This paper provides direct evidence that dopamine secreted by the brain directly into the systemic circulation in this period of ontogenesis has an inhibitory effect on prolactin secretion by pituitary cells. These results provide the basis for a fundamentally new understanding of the brain’s role in the neuroendocrine regulation of the development and function of peripheral target organs and, particularly in this study, the pituitary gland.

## INTRODUCTION

In adult animals, the brain, and particularly the hypothalamus, is the central part of the neuroendocrine system responsible for the regulation of the most important functions and maintenance of a constant internal environment for the body. Of particular interest is the formation and function of the neuroendocrine regulatory system in ontogenesis, since hypothalamic neurohormones and hormones of the endocrine glands regulate not only the functional activity of target organs, but their development during the development of an organism, as well. In the latter case, the action of these signaling molecules is of irreversible (imprinting- like, morphogenetic) nature [3-3]. According to the concept established by the end of the 1980s, the brain is not involved in neuroendocrine regulation of peripheral organs until its full maturity; i.e., the formation of interneuron synaptic connections and blood-brain barrier (BBB). It is only after the final differentiation of neurons and establishment of synaptic neurotransmission that it takes control over the pituitary gland and all the other endocrine glands through it [4, 5].

In recent years, our understanding of the brain’s role in neuroendocrine regulation in ontogenesis has undergone significant changes. After an analysis of published data and the results of our own research in our laboratory, we noticed that neurons begin to synthesize and secrete signaling molecules long before the formation of interneuron synaptic connections and BBB [6-8]. This observation allowed us to put forth the hypothesis that, prior to BBB formation, the brain functions as an endocrine organ releasing physiologically active substances into the systemic circulation and, thus, affecting the development and function of peripheral organs and target cells [8].

The two pillars of this hypothesis are that

1) The brain is the source of signaling molecules in the systemic circulation from the moment of neuron formation to the final formation of synaptic connections and BBB closure and

2) Signaling molecules secreted by the brain into the systemic circulation during that period of ontogenesis can have a direct para-adenohypophyseal endocrine effect on peripheral target organs.

The first leg of the hypothesis was confirmed by the studies performed in our laboratory. Direct evidence that during the early postnatal period prior to BBB formation the brain serves as the source of dopamine in the systemic circulation, was demonstrated in the model of specific reversible inhibition of the synthesis of catecholamines in the brain of neonatal animals [9].

When testing the validity of the second part of the hypothesis, with *in vitro *and *ex vivo *experiments in our laboratory, the obtained data showed that dopamine has an inhibitory effect on prolactin production by pituitary cells at the concentration at which it is present in the systemic circulation [10].

In adult animals, neuroendocrine regulation is carried out mainly through the hypothalamic-hypophyseal portal circulation system. In the absence of BBB during the perinatal period of ontogenesis in rats, the regulation can technically be carried out both through portal and systemic circulation. So far, however, no data has been obtained on the contribution of each of the pathways to the neuroendocrine regulation of the function and development of peripheral organs. The answer to this question is of fundamental importance not only for the confirmation of the endocrine function of the brain, but also for understanding the development and function of the neuroendocrine regulation system in ontogenesis.

In this regard, the purpose of our research was to study the effect of dopamine secreted by the brain into the systemic circulation on the synthesis of prolactin by the pituitary gland in ontogenesis. Using 6-hydroxydopamine neurotoxin in the model of a chronic specific shutdown of dopamine synthesis in the brain of neonatal rats, we tried to assess

1) the morphological and functional state of dopaminergic neurons of the tuberoinfundibular system of the brain;

2) the endocrine effect of brain-produced dopamine present in the systemic circulation on the synthesis and release of prolactin by pituitary cells.

## EXPERIMENTAL SECTION

**Animals, experiments**

**Fig. 1:**
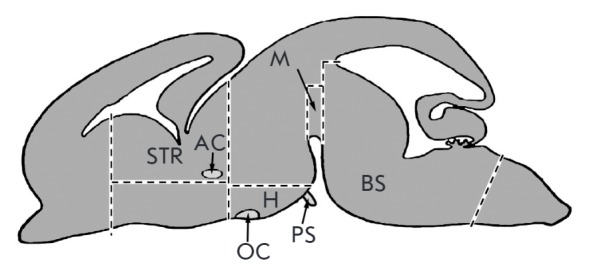
Scheme of brain structures isolation after the administration of neurotoxin. STR – striatum, AC – anterior commissure, H – mediobasal hypothalamus, PS – pituitary stalk, M –midbrain, BS – brain stem, OC – optic chiasm

We used 90 Wistar male rats in the second day of postnatal development (P2). In order to obtain a model of chronic specific inhibition of DA synthesis in the brain, 100 μg of 6-hydroxydopamine (6-OHDA, Sigma, USA) was stereotaxically injected into the lateral ventricles of the brains of the rats, while control animals were injected with 0.9% NaCl [11]. In order to effect selective destruction of dopaminergic neurons and preserve noradrenergic neurons, 25 mg/kg of desmethylimipramine (DMI), an inhibitor of noradrenaline and 6-OHDA reuptake into noradrenergic neurons, was injected subcutaneously 30 min prior to the administration of 6-OHDA. 72 h after 6-OHDA injection brain sections were isolated in anesthetized rats (chloral hydrate, 400 mg/kg, Sigma, USA) as shown in *Fig. 1*. Samples were frozen in liquid nitrogen and stored at –70°C prior to high-performance liquid chromatography with electrochemical detection (HPLC-ED).

To determine tyrosine hydroxylase (TH) activity, all the animals (experimental and control) were intraperitoneally injected with the aromatic *L-*amino acid decarboxylase (AAAD) inhibitor: 3-hydroxybenzyl hydrazine (NSD-1015, Sigma, USA) at a concentration of 100 mg/kg of body weight 72 hours after stereotactic injection of 6-OHDA and 30 minutes prior to obtaining the samples [12]. Next, mediobasal hypothalamus and the rest of the brain were isolated under anesthesia. TH activity in the collected samples was assessed by accumulation of *L*-dihydroxyphenylalanine (*L-*DOPA) measured by HPLC-ED.

The number of mono- and bienzymatic neurons in the arcuate nucleus of the rats was assessed by immunohistochemistry. For this purpose, transcardial perfusion was carried out first with 0.02 M phosphate-buffered saline (PBS, pH 7.2–7.4) for 10–15 minutes and then with 4% pre-cooled (to +4°C) paraformaldehyde in 0.2 M phosphate buffer (pH 7.3) for 15 min. Then, the brain was isolated and postfixation with 4% paraformaldehyde was performed at +4°C for 12 h. After that, the brain was washed in 0.02 M PBS (3 times for 15 min each), incubated in 20% sucrose at +4°C for 24 hours and frozen in hexane at –40°C. Prior to the immunohistochemical analysis, the samples were stored at –70°C.

Blood collected from the heart of anesthetized animals was centrifuged (7,000 rpm, 20 min, +4°C), and then the prolactin (PRL) level in the blood plasma was measured by ELISA.

When determining PRL in pituitary tissue, the hormone was first extracted with 0.05 M carbonate-bicarbonate buffer [10].

The PRL mRNA content was measured in the anterior part of the pituitary gland isolated after stereotactic injections. Each sample contained material obtained from three rats. Prior to RNA isolation, the samples were stored at –70°C.

**High-performance liquid chromatography**

The content of catecholamines and metabolites in brain tissue was determined by reverse phase HPLC with electrochemical detection [11].

**Immunohistochemistry**

TH and AAAD were detected in 20-μm sections of mediobasal hypothalamus prepared on a cryostat and mounted onto the glass. Sections were sequentially incubated with (a) 3% bovine serum albumin (Sigma, USA) and 0.3% Triton X-100 (Sigma, USA) in 0.02 M PBS for 30 min at +20°C; (b) 1% sodium lauryl sulfate (SDS, Sigma, USA) in 0.02 M PBS for 3 min at +20°C; (c) sheep polyclonal antibodies to TH (1 : 700) (Chemicon, USA) and rabbit polyclonal antibodies to AAAD (1 : 300) (Abcam, USA) in 0.02 M PBS containing 1% bovine serum albumin and 0.1% Triton X-100 at +20°C for 24 hours; (d) FITC-conjugated donkey antibodies to sheep gamma globulins (1 : 40) (FITC antisheep, Sigma, USA) and Cy3-conjugated goat antibodies to rabbit gamma globulins (1 : 500) (CY3 antirabbit, Sigma, USA ) in 0.02 M PBS at +20°C for 2 hours. After each incubation, except for the last one, the sections were washed in 0.02 M PBS for 15 min. After the final incubation, the sections were washed in 0.02 M PBS for 1 h and then embedded into the hydrophilic medium Mowiol (Calbiochem, Germany).

Slices of hypothalamus after double labeling to TH and AAAD were examined using a fluorescence microscope Zeiss Observer Z1 equipped with filters for FITC (for TH) and Cy3 (for AAAD) using the AxioVision 4.8 software.

## ELISA

The PRL content in the tissue of the anterior part of the pituitary gland and plasma samples was determined by ELISA using commercial kits SPIbio-Rat Prolactin EIA Kit (Bertin Pharma, France).

**Real-time PCR**

Total RNA was isolated using TRI Reagent (Sigma, USA) according to the manufacturer’s protocol. In order to remove contaminants of genomic DNA, the isolated RNA was treated with DNase (Fermentas, USA). RNA was reprecipitated in 4 M LiCl, and RNA concentration was measured using a Nanodrop 8000 spectrophotometer (Thermo Scientific, USA). cDNA was synthesized using reverse transcriptase M-MLV and hexameric oligonucleotides (Fermentas, USA).

Real-time PCR was performed in an automated thermocycler 7500 Real-Time PCR System (Applied Biosystems, USA) using a qPCRmix-HS SYBR+ROX mixture (Fermentas, USA) and specific oligonucleotides (“Lytech”, Russia). Sense sequence: 5’-ATAGATGATTGGGAGGGGAAGAG- 3’; antisense sequence: 5’-CATCATCAGCAGGAGGAGTGTC-3’. The values obtained for each sample were normalized to the expression of the household gene *GAPDH*. The *GAPDH* gene expression level was determined using primers: sense – 5’-CTGACATGCCGCCTGGAGAAA-3’; antisense – 5’-TGGAAGAATGGGAGTTGCTGTTGA-3’.

Relative gene expression was calculated by the ΔΔCt method taking into account PCR efficiency. PCR efficiency was determined by the construction of standard curves [13].

**Statistical analysis**

Analysis of statistical data was performed using the integrated GraphPad Prism Version 6.0 software for Windows (GraphPad Software, USA). Data are shown as mean ± SEM (M ± m). The statistical significance of the results was determined using the parametric Student’s* t*-test (*t*-test) and nonparametric Mann-Whitney U-test (U-test).

## RESULTS

**Concentration of dopamine in various brain regions**

**Fig. 2:**
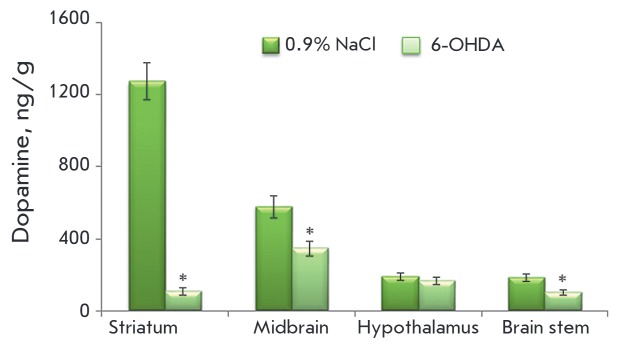
Concentration of dopamine in various brain regions 72 hours after stereotactic injection of 100 μg of 6-OHDA into the lateral ventricles of the brain following subcutaneous administration of 25 mg/kg DMI; control was injected with 25 mg/kg DMI and 0.9% NaCl. * – Statistically significant differences between the control and the experiment

Seventy-two hours after 6-OHDA administration, the striatal DA concentration decreased by 92%, and by 40% and 44% in the midbrain and brain stem, respectively. At the same time, the concentration of DA in the hypothalamus did not change compared to the control (*Fig. 2*).

**TH activity in various brain regions**

**Fig. 3:**
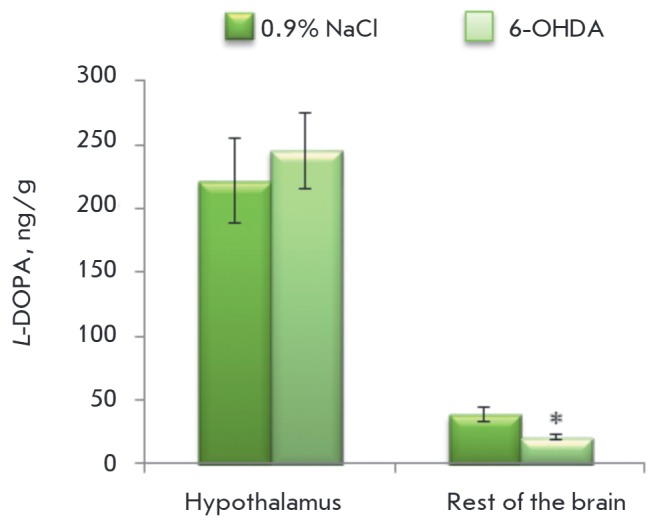
Tyrosine hydroxylase activity assessed by the accumulation of dihydroxyphenylalanine (*L*-DOPA) 30 minutes after the injection of 3-hydroxybenzylhydrazine (NSD-1015) in the hypothalamus and the rest of the brain on the model of chronic inhibition of dopamine synthesis in the brain with 6-OHDA. * – Statistically significant differences between the control and the experiment

TH activity was evaluated by the accumulation of* L*-DOPA after the administration of the AAAD inhibitor NSD-1015. When modeling the deficiency of DA synthesis in the brain of neonatal rats, the *L-*DOPA concentration in the hypothalamus did not change compared to the control, while a statistically significant reduction was observed in the rest of the brain (*Fig. 3*).

**Number of mono- and bienzymatic neurons in the arcuate nucleus**

**Fig. 4:**
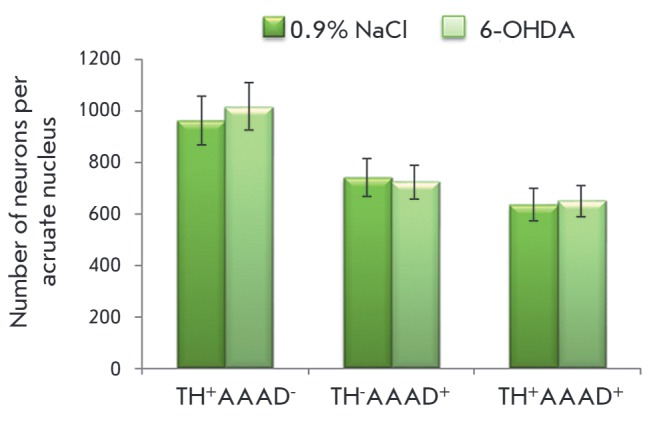
Number of monoenzymatic TH^+^ AAAD^-^, monoenzymatic TH^-^ AADC^+^ , and bienzymatic TH^+^ AAAD^+^ neurons in the arcuate nucleus 72 hours after the administration of 100 mg of 6-OHDA into the lateral ventricles of the brain following systemic administration of 25 mg/kg DMI; the control was injected with 25 mg/kg DMI and 0.9% NaCl

**Fig. 5:**
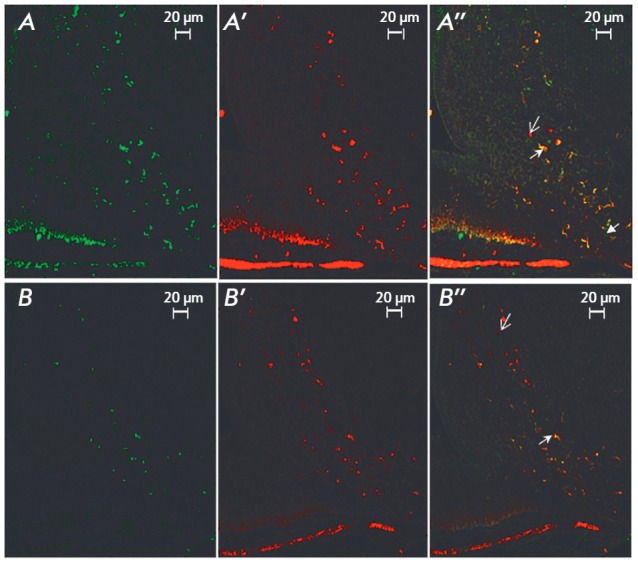
Fluorescence microscopy. Images of tyrosine hydroxylase positive (*A, B*), aromatic amino acids decarboxylase positive (*A’, B’*), and tyrosine hydroxylase and aromatic amino acids decarboxylase positive (*A’’, B’’*) neurons in the arcuate nucleus in the control (*A, A’, A’’*) and after stereotactic injection of 6-OHDA (*B, B’, B’’*). Arrows indicate neurons of different phenotypes

Seventy-two hours after 6-OHDA administration into the lateral ventricles of the rats, the number of monoenzymatic TH^+^AAAD^-^, monoenzymatic TH^-^AAAD^+^, and bienzymatic TH^+^AAAD^+^ neurons in the arcuate nucleus had not change compared to the control (*Fig. 4*, *Fig. 5*).

**Prolactin in pituitary gland and blood plasma**

**Fig. 6:**
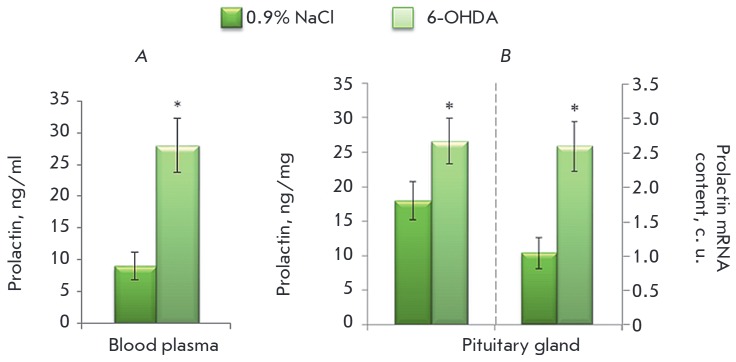
Concentration of prolactin in the blood plasma (*A*) and concentration of prolactin and prolactin mRNA content in the pituitary gland (*B*) 72 hours after the administration of 100 μg of 6-OHDA into the lateral ventricles of the brain following systemic administration of 25 mg/kg DMI; the control was injected with 25 mg/kg DMI and 0.9% NaCl. * – Statistically significant differences between the control and the experiment

Seventy-two hours after the introduction of 100 μg of 6-OHDA into the lateral ventricles of the rats, the prolactin concentration increased in a statistically significant manner by 70% and 48% in the blood plasma and pituitary gland, respectively. At the same time, the prolactin mRNA content in the pituitary gland increased 2.5-fold (*Fig. 6*).

## DISCUSSION

In adult animals, DA transferred from the hypothalamus to the pituitary gland regulates the function of peripheral targets only through the portal circulation system, whereas, according to our hypothesis, DA, in the absence of BBB in neonatal animals, can be also transferred from all populations of neurons directly into the systemic circulation. We have earlier shown in *in vitro* experiments that dopamine, at the concentration at which it is present in the peripheral blood of neonatal rats, inhibits prolactin secretion by the pituitary gland [10, 14]. However, pituitary cells are under the constant tonic influence of DA under *in vivo *conditions, while in* in vitro *conditions, the pituitary cells are contained in a medium without DA for a long period of time, which, according to some scientists, can fundamentally change cell physiology [15]. Moreover, this approach does not allow one to say for certain whether the regulation is carried out through the systemic circulation or not, since the concentration of DA in peripheral and portal blood at this stage of ontogenesis can be comparable.

Analysis of the published data allows us to suggest that this problem can be solved by inhibiting DA synthesis in the brain using 6-OHDA-specific neurotoxin of CAergic neurons, which enters the cell via specific transporters of DA and norepinephrine and inhibits the process of oxidative phosphorylation [16].

Due to the fact that the expression level of the membrane transporter of dopamine in mediobasal hypothalamus neurons is rather low and that uptake mechanisms in neonatal animals are undeveloped [17, 18], we hypothesized that the use of 6-OHDA in the modeling of DA deficiency in the brain of neonatal rats would allow us to divide the above-described pathways of regulation of the functional activity of pituitary cells. In this regard, we have developed a model of specific inhibition of dopamine synthesis in the brain of newborn rats. In this model, the dopamine level in the brain fell by 54%, while the decrease was even more dramatic in the blood plasma (70%) [11]. On the basis of the available data, one cannot draw an unambiguous conclusion that the effects that are seen in this model are provided only by DA transferred from the brain into the systemic circulation. In order to assess whether the neurotoxin affects DA-producing mediobasal hypothalamic neurons, we first identified the content of DA and its metabolites in this region after 6-OHDA administration. It has been shown that, in our model, the DA level in mediobasal hypothalamus does not change compared to the control, while it is statistically significantly reduced in the striatum, midbrain, and brain stem. These data can be regarded as an integral indicator that the secretory activity of the hypothalamus does not change under the effect of the toxin.

DA is synthesized from amino acid tyrosine by two enzymes: TH and AAAD. For this reaction chain, the rate-limiting step is the synthesis of *L*-DOPA from tyrosine under the action of TH [19]. Therefore, we evaluated TH activity by determining the accumulation of* L*-DOPA in the mediobasal hypothalamus after the inhibition of the second enzyme of DA – AAAD synthesis as a direct indicator of DA synthesis in our model. It was found that the *L*-DOPA level did not change in this region compared to the control; i.e., TH activity did not change under the action of neurotoxin. At the same time, the concentration of *L*-DOPA in the rest of the brain was reduced 2-fold compared to the control.

The rate of brain neuron degradation under the influence of 6-OHDA was evaluated mainly by immunolabeling for TH. However, it is known that in addition to true DAergic neurons expressing both enzymes of DA synthesis, there are also neurons containing only one of the enzymes [20-22]. It has been previously shown in our laboratory that the number of monoenzymatic neurons in the arcuate nucleus of rats during the perinatal period is much higher than the number of bienzymatic neurons. Thus, the number of monoenzymatic neurons in this region at E21 is 99%, while the number of bienzymatic neurons is only 1%. At the P9 stage, bienzymatic neurons constitute 38% [23]. Furthermore, it was shown that monoenzymatic neurons are able to carry out a cooperative synthesis of DA [24, 25]. In addition to this, in the case of functional insufficiency of the hypothalamic tuberoinfundibular system caused by the introduction of 6-OHDA into the brain of adult animals, the number of both TH- and AAAD-containing monoenzymatic neurons increases [26]. Apparently, such a reaction is a manifestation of compensatory processes. In this regard, we evaluated the number of neurons in the arcuate nucleus by double immunolabeling for TH and AAAD in the DA deficiency model. It turned out that the amount of bienzymatic, monoenzymatic TH-containing and monoenzymatic AAAD-containing neurons did not change compared to the control.

Thus, we have obtained evidence that specific inhibition of DA synthesis in the brain with the 6-OHDA neurotoxin does not change the morphological and functional state of the mediobasal hypothalamus. This implies that, if changes in PRL synthesis are detected in this model, they are due to the influence of DA, exclusively, via the systemic circulation.

In the next series of experiments, we evaluated the effect of DA secreted by the brain into the systemic circulation on the synthesis of prolactin by the pituitary gland. It has been previously shown in our laboratory that the inhibitory effect of DA on the secretion of prolactin is first detected at the E21 stage [27]; i.e., this mechanism should be sufficiently mature in the analyzed period. It was found that the PRL concentration increased by 70% upon a decrease in DA in the plasma of 73%. This indicator characterizes the level of PRL secretion by pituitary cells. Such a significant increase in PRL concentration in the plasma in response to the decrease in DA concentration implies that dopamine secreted by the brain into the systemic circulation has an inhibitory effect on the secretion of PRL by pituitary cells. Moreover, the concentration of PRL in the pituitary gland increases by 48%. Apparently, DA entering the pituitary gland through the systemic circulation does not only inhibit PRL secretion, but also affects its synthesis. In order to confirm the effect of DA on PRL synthesis in our model, we evaluated the level of PRL mRNA. It was found that the level of PRL mRNA expression in the pituitary gland of animals deficient in DA is 2.5-fold higher than that in the control.

## CONCLUSION

Thus, we were able to show that DA secreted by the brain into the systemic circulation has an inhibitory effect on the synthesis and secretion of PRL by the pituitary cells in the early postnatal period prior to BBB formation. According to our hypothesis on the endocrine function of the brain before BBB formation in the early postnatal period, there are two pathways of dopamine regulation of PRL secretion by the pituitary gland: through DAergic neurons of the hypothalamus via the portal circulation system and through other populations of DAergic neurons via the systemic circulation. It can be assumed that during the period of ontogenesis that we studied, the regulation of the pituitary cell function through systemic circulation significantly contributes to the regulation of PRL secretion.

## Abbreviations

HPLC: high-performance liquid chromatography
BBB: blood-brain barrier
6-OHDA: 6-hydroxydopamine
DA: dopamine
AAAD: aromatic L-amino acid decarboxylase
PRL: prolactin
TH: tyrosine hydroxylase
L-DOPA: L-dihydroxyphenylalanine
